# Serological detection of ‘*Candidatus* Liberibacter asiaticus’ in citrus, and identification by GeLC-MS/MS of a chaperone protein responding to cellular pathogens

**DOI:** 10.1038/srep29272

**Published:** 2016-07-06

**Authors:** Fang Ding, Yongping Duan, Qing Yuan, Jonathan Shao, John S. Hartung

**Affiliations:** 1 Hubei Key Laboratory of Plant Pathology, Huazhong Agricultural University, Wuhan 430070 P. R. China; 2 USDA ARS MPPL 10300 Baltimore Ave., Beltsville, MD 20705 USA; 3 USDA ARS USHRL 2001 South Rock Road, Fort Pierce, FL 34945 USA; 4 Sichuan Medical University, Luzhou, Sichuan, 646000 P. R. China

## Abstract

We describe experiments with antibodies against ‘*Candidatus* Liberibacter asiaticus used to detect the pathogen in infected plants. We used scFv selected to bind epitopes exposed on the surface of the bacterium in tissue prints, with secondary monoclonal antibodies directed at a FLAG epitope included at the carboxyl end of the scFv. Unexpectedly, the anti-FLAG secondary antibody produced positive results with *C*aLas diseased samples when the primary scFv were not used. The anti-FLAG monoclonal antibody (Mab) also identified plants infected with other vascular pathogens. We then identified a paralogous group of secreted chaperone proteins in the HSP-90 family that contained the amino acid sequence DDDDK identical to the carboxy-terminal sequence of the FLAG epitope. A rabbit polyclonal antibody against one of the same epitopes combined with a goat anti-rabbit secondary antibody produced very strong purple color in individual phloem cells, as expected for this pathogen. These results were entirely specific for *C*aLas-infected citrus. The simplicity, cost and ability to scale the tissue print assay makes this an attractive assay to complement PCR-based assays currently in use. The partial FLAG epitope may itself be useful as a molecular marker for the rapid screening of citrus plants for the presence of vascular pathogens.

Huanglongbing (HLB), also known as citrus greening disease, poses an existential threat to the citrus industry in the United States^1^. The disease is associated with a non-cultured member of the α-proteobacteria, ‘*Candidatus* Liberibacter asiaticus’ (*C*aLas)^2^. The pathogen has long been present in southeastern China^3^ but the first unambiguous description of HLB was in India^4^, and since that time HLB has been a limiting factor for citriculture in Asia, but not in the new world where it was unknown^5^. The pathogen is transmitted to and among citrus trees by the Asian citrus psyllid (ACP), *Diaphorina citri* Kuwayama^6^, which in the absence of *C*aLas causes only minor feeding damage as it reproduces and feeds on citrus trees. The ACP was reported in Brazil in the 1950’s but HLB was not reported there until 2004^7^,^8^. The ACP was reported in Florida in 1998 and rapidly established itself throughout the Caribbean^9^, and HLB was found to be endemic in Florida in 2005^5^. The bacterium colonizes both the citrus plant and psyllid systemically and persistently. The psyllid feeds on citrus phloem in the leafy portion and young stems of the trees. All commercial varieties of citrus are susceptible, and infected trees enter a progressive decline. Although genetically modified citrus with useful levels of resistance to CaLas have recently been developed^10^, and progress has been made towards potentially therapeutic strategies^11^, huanglongbing remains an extraordinary challenge to the global citrus industry. Current best management practices are based on suppression of the ACP population with area-wide application of a pesticide program to limit disease spread, and the application of aggressive plant nutrition programs to support production from infected trees^12^,^13^.

The biology of *C*aLas and citrus poses several problems for research on HLB. *C*aLas has not been cultured *in vitro*, despite many attempts, and this has limited methods for detection and quantification of the bacterium to PCR and qPCR. The population level of *C*aLas in the tree is very variable, and is not well correlated with foliar symptoms, which are themselves variable^14^,^15^. Examination of infected plant tissue with light microscopy shows disruption and plugging of the phloem and ultrastructural studies have shown that adjacent cells in the phloem can be completely filled with *C*aLas or empty^16^,^17^. Recent work has drawn attention to the importance of early colonization of the roots of citrus trees after infection^18^,^19^.

Antibodies have long been a mainstay in the study of plant pathogens, and are applied to detect, identify and quantify pathogens. Because *C*aLas has not been cultured, conventional polyclonal antibodies raised against the whole bacterial cell have not been produced. However the genome of *C*aLas has been determined^20^, which makes proteins of *C*aLas available for use as antigens by PCR-based cloning. We have recently developed a library of antibodies against *C*aLas that can be screened to isolate antibodies against any defined and cloned *C*aLas antigen. This library of single chain antibodies (scFv) was made using phage display technology, and includes unique mouse antibodies that recognize antigens present in psyllids infected by *C*aLas that were used to immunize mice as the first step in the creation of the library. scFv are available that recognize antigens expressed on the surface of *C*aLas cells, including the major outer membrane protein, OmpA and a protein component of the basal flagellar apparatus, FlgI^21^.

Tissue printing is a well-established application of antibodies for plant research. Sections of plant tissue are pressed onto a membrane, typically nitrocellulose, and a faithful image of the cut surface of the plant tissue is bound to and preserved on the nitrocellulose membrane. Antibodies can then be used to detect and visually describe the distribution of antigens of interest in these tissue prints. Tissue printing was first used to study the distribution of the cell wall protein extensin in soybean plants^22^. The anatomical detail of the plant is preserved in tissue prints, and the technique has also been used to study the distribution of plant viruses in infected plants^23^ and in particular a direct tissue blot immunoassay (DTBIA) has been used for the detection of *Citrus tristeza virus* (CTV)^24^. As noted above, qPCR has been used to study the distribution of *C*aLas in citrus, but anatomical information is destroyed in the processing of the samples. Thus DTBIA, which is a relatively simple and inexpensive technique, is ideally suited to study the distribution of *C*aLas with anatomical detail in infected citrus. We have tested scFv in DTBIA assays for *C*aLas in infected sweet orange and rough lemon trees. The method used secondary monoclonal antibodies directed at a FLAG epitope included at the carboxyl end of the scFv^25^ for detection of the antibodies bound to the tissue prints. We also used a polyclonal antibody (Pab) produced in a rabbit against the major outer membrane protein, OmpA, in tissue print assays of similar plant tissues. The Pab was in turn detected by a goat anti-rabbit IgG polyclonal antibody. This allowed a direct comparison between the polyclonal and scFv recombinant antibodies for DTBIA.

## Results

### Detection of *Ca*Las with ScFv antibodies by direct tissue blot immuno assay (DTBIA)

Single chain variable fragment antibodies scFv 748 (OmpA) and scFv 932 (FlgI) were selected for testing in the DTBIA. These scFv had been used successfully when expressed from phagemid vector pKM19 to detect purified protein antigens and in dot blot assays against crude extracts from infected sweet orange^21^. In the present work the scFv were expressed from the plasmid vector pKM16 in *E. coli* BL21 and purified by taking advantage of the 6X His tag provided by the vector. The scFv preparations were shown to be pure by SDS-PAGE and successfully detected their purified antigenic targets in dot blot and western blot assays (data not shown).

#### Recognition of CaLas antigen by ScFv antibody in citrus samples

**Figure 1:**
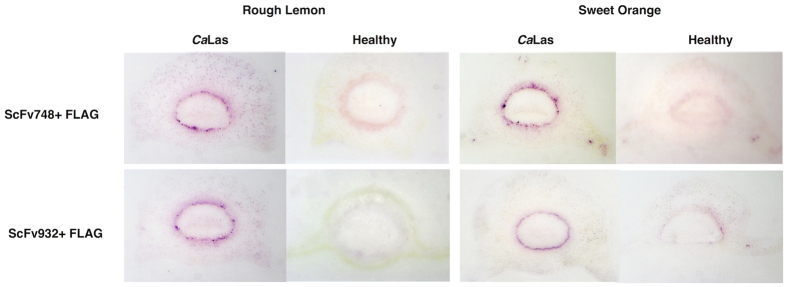
Tissue prints were made for DTBIA from petioles of *C*aLas infected and healthy rough lemon and sweet orange trees. scFv748 and scFv932 were used as primary antibodies (1:1000 dilution) in the top and bottom rows, respectively. Secondary anti-FLAG Mab conjugated with alkaline phosphatase was used (1:10,000 dilution). Detection was with NBT/BCIP.

Petioles of sweet orange and rough lemon leaves from HLB-symptomatic trees were used to prepare tissue prints to detect *C*aLas using the FLAG epitope incorporated into the scFv and a secondary anti- FLAG Mab conjugated with alkaline phosphatase. All petioles used for DTBIA were also tested by qPCR after the tissue prints had been prepared. Typical results of qPCR assays of the petiole sections used for tissue printing showed that the concentration of *Ca*Las DNA was consistently high in these petioles, typically with a Cq value of ~21 (Table 1). This represents about 10^7^ *Ca*Las genomes per g of petiole^26^. The results of the DTBIA appeared to show that scFv 748 and scFv932 specifically recognized *C*aLas in DTBIA from petioles of rough lemon and sweet orange. Color development was strong and limited to only some cells in the phloem ring in infected petioles of both rough lemon and sweet orange as expected for the phloem limited pathogen (Fig. 1). Diffuse color development, not focused in the phloem cells, was often observed in the vascular ring of the healthy samples as well, though the diseased and healthy samples could easily be distinguished.

### Identification of a protein with a partial FLAG epitope in response to cellular pathogens

**Figure 2:**
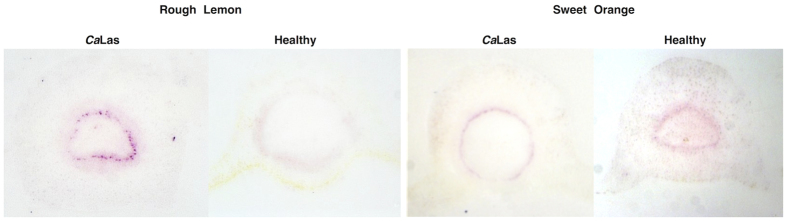
Tissue prints were made for DTBIA from petioles of CaLas infected and healthy rough lemon and sweet orange trees. Tissue prints were developed with only the secondary anti-FLAG antibody. No primary scFv labeled with FLAG were used. The secondary anti-FLAG Mab (Sigma) was conjugated with alkaline phosphatase and used at 1:10,000 dilution. Detection was with NBT/BCIP.

Further experiments were carried out to optimize the concentration of scFv and detection antibody in these experiments, and the results looked promising, with strong purple color in the phloem sieve cells of *C*aLas infected, but not healthy petioles. As a control, the secondary anti-FLAG Mab was incubated with tissue prints of *C*aLas infected petioles without the use of any primary scFv that contained the FLAG epitope. Surprisingly, the results with the secondary Mab alone were difficult to distinguish from those when both the primary scFv and secondary Mab were used together (Fig. 2). Color development was sharply localized in phloem sieve cells of *C*aLas infected petioles and more weakly and without sharp foci in the area of the vascular rings of the healthy plant controls.

#### A protein with a partial FLAG epitope in citrus responds to other cellular pathogens

**Figure 3:**
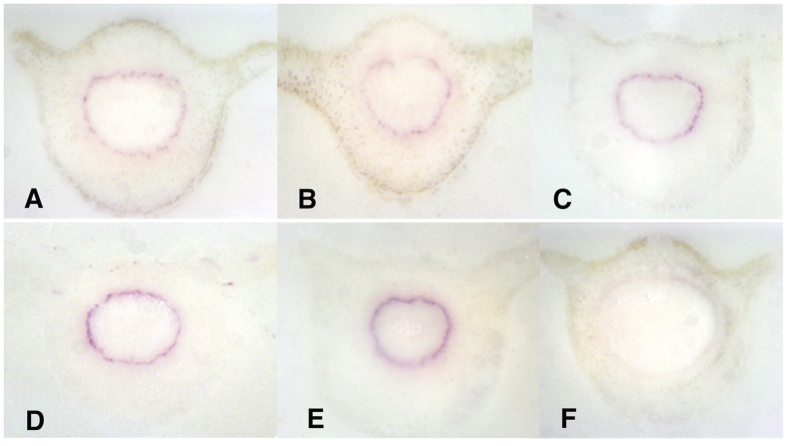
DTBIA for vascular limited citrus pathogens. A secondary anti-FLAG antibody conjugated with alkaline phosphatase was used at 1:10,000 dilution without any primary scFv antibodies for specific pathogen detection. (**A**) CTV B2 in sweet orange (**B**) CTV B6 in sweet orange (**C**) CCDV in *Citrus macrophylla* (**D**) CaLas in sweet orange (**E**) X. *fastidious* in sweet orange (**F**) Healthy sweet orange. Detection was with NBT/BCIP.

**Figure 4:**
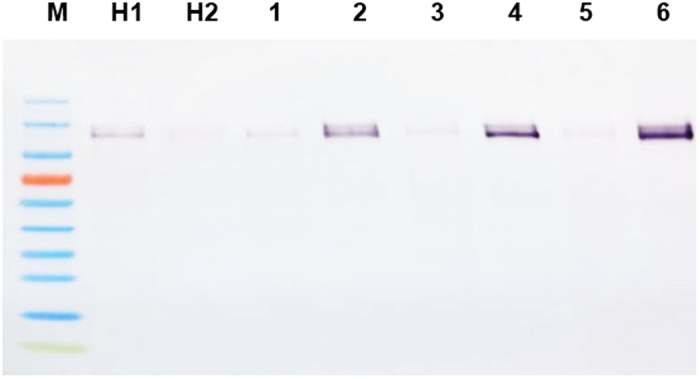
FLAG-like epitope in *C. sinensis* new flush (NF) and mature leaves (ML) confirmed by western blot as compared in healthy and disease leaves by different kind of citrus cellular pathogens. H1 and H2: Healthy new flush (NF) and healthy mature leaves (ML); CaLas-infected NF (1) and ML(2); *Citrus tristeza virus* infected NF(3) and ML(4); Citrus chlorotic dwarf virus infected NF(5) and ML(6). Total protein was adjusted to same concentration with standard protein marker (Dojindo, technologies), 15 ug was loaded for each well.

Because the anti-FLAG secondary Mab appeared to recognize protein in the phloem tissue in petioles from HLB-diseased trees, we tested petioles from trees infected with other phloem and xylem limited pathogens. Similar results were obtained when petioles infected with two genotypes of CTV, as well as with CCDV and *Xylella fastidiosa*. Strong color developed in the vascular ring of petioles from mature leaves from the infected, but not from the healthy control plants, with sharp foci in the phloem cells (Fig. 3). A protein with a partial FLAG epitope was also shown to be present in infected leaves of C*. sinensis* by western blot analysis of proteins extracted from both diseased and healthy leaves (Fig. 4). These results showed that the protein with the partial FLAG epitope was not detectable in healthy, mature citrus leaves, though it was weakly expressed in healthy, immature citrus leaves. However, the protein with a partial FLAG epitope was strongly expressed in leaves infected by CaLas, CTV or CCDV.

#### Identification of the partial FLAG epitope

A list of *C. sinensis* peptides expressed in diseased petioles of mature leaves was compiled by massively parallel identification of peptides by GeLC-MS/MS. An automated p-BLAST search of the *C. sinensis* proteome identified several peptides that were portions of a paralogous group of *C. sinensis* proteins (Table 2). The full-length proteins shared a common amino acid sequence of DDDDK, the carboxy terminal five amino acids of the FLAG octapeptide. A similar search of the *C. clementina* proteome returned a similar set of proteins (Table 3; Figure S2). The paralogous proteins from *C. sinensis* were all members of the heat shock protein-90 (HSP-90) family of proteins.

### Detection of CaLas with an anti-OmpA polyclonal antibody by DTBIA

**Figure 5:**
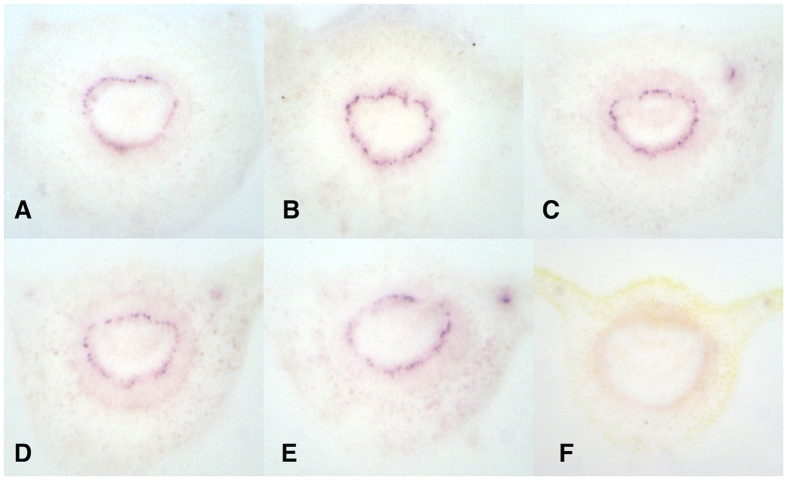
DTBIA for the detection of *Ca*Las in sweet orange petioles using rabbit polyclonal antibody raised against the major outer membrane protein OmpA. The primary antibody (1:5000 dilution) was detected with Goat anti-rabbit secondary Pab diluted 1:50,000. (**A–E**) *Ca*Las B430 in different sections of petioles. (**F**) Healthy Sweet orange. Detection was with NBT/BCIP.

**Figure 6:**
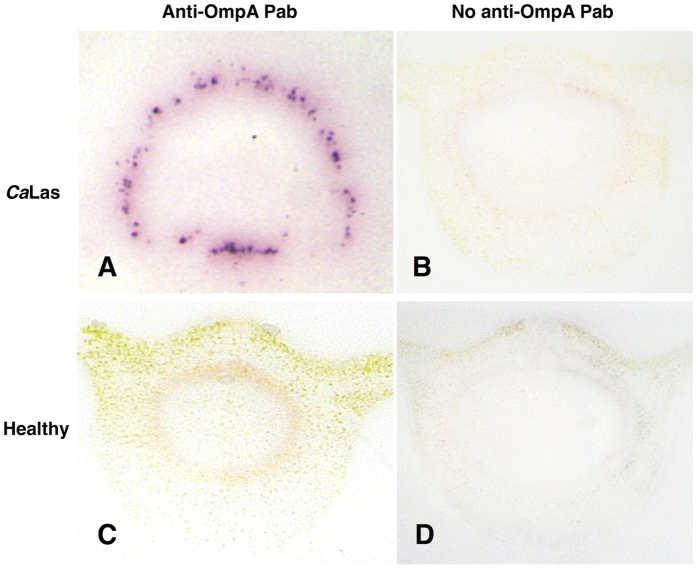
DTBIA of *C*aLas infected and healthy sweet orange petioles. Tissue prints were probed with (**A,C**) polyclonal rabbit anti-OmpA antibodies and goat anti rabbit secondary Pab or (**B,D**) with the goat anti-rabbit secondary Pab alone. (**A,B**) *C*aLas infected sweet orange. (**C,D**) healthy sweet orange.

**Figure 7:**
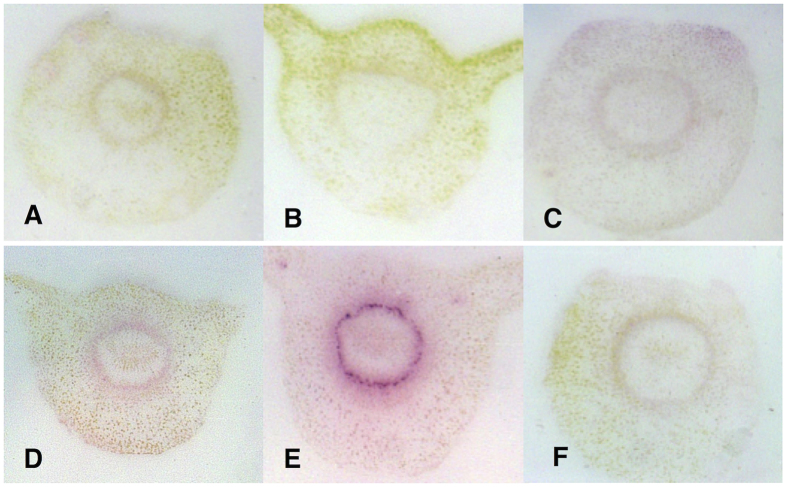
Specificity of the rabbit polyclonal anti-OmpA antibody in DTBIA of citrus petioles. (**A**) Sweet orange infected by CTV B2. (**B**) Sweet orange infected by CTV B6. (**C**) *C. macrophylla* infected with CCDV. (**D**) Sweet orange infected with *X. fastidiosa*. (**E**) *Ca*Las infected sweet orange. (**F**) Healthy sweet orange. The primary antibody (1:5000 dilution) was detected with Goat anti-rabbit secondary Pab diluted 1:50,000.

**Figure 8:**
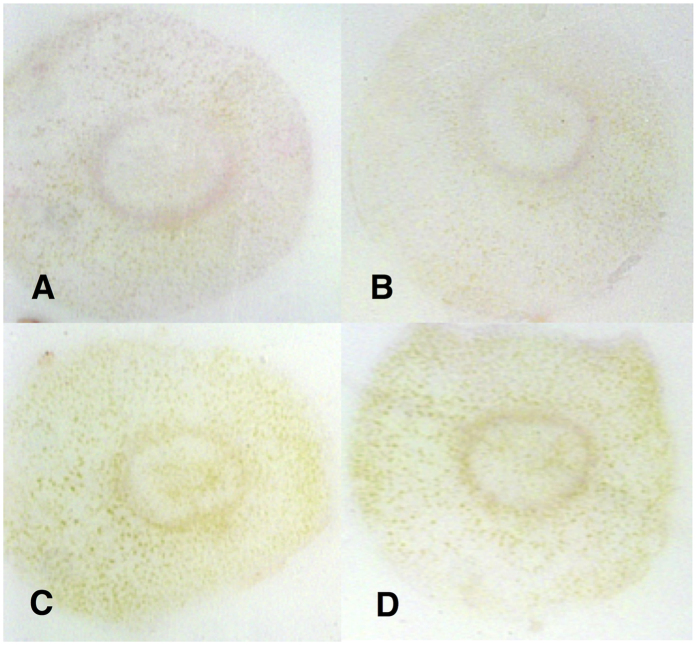
Specificity of the secondary goat anti-rabbit Pab in DTBIA of citrus petioles. (**A**) Sweet orange infected by CTV B2. (**B**) Sweet orange infected by CTV B6. (**C**) *C. macrophylla* infected with CCDV. (**D**) Sweet orange infected with *X. fastidiosa*. The polyclonal rabbit anti OmpA antibody was not used; only the goat anti rabbit secondary Pab conjugated with alkaline phosphatase was used. Detection was with NBT/BCIP.

We also tested a polyclonal rabbit antibody (Pab) prepared against the same portion of the major outer membrane protein of *C*aLas, OmpA. The anti-OmpA Pab was then detected with a secondary goat anti rabbit Pab conjugated with alkaline phosphatase. When this antibody system was used for DTBIA from *C*aLas infected and healthy sweet orange petioles, strong color foci were observed in the phloem sieve cells, as expected for *C*aLas, without similar color development in the DTBIA from petioles of healthy sweet orange (Fig. 5). When the secondary goat anti-rabbit Pab was used without the primary rabbit anti OmpA antibody, no color was observed in the immuno tissue prints (Fig. 6). The specificity of the polyclonal rabbit anti-OmpA antibody was tested against the panel of phloem-limited citrus pathogens. Color in the vascular ring was observed in DTBIA of petioles from trees infected with *C*aLas, but not in DTBIA of petioles from trees infected with phloem limited viruses *or Xylella fastidiosa* (Fig. 7). Purple color in the phloem sieve tubes also was not observed in the phloem sieve cells in the DTBIA prepared from petioles from trees infected with the other phloem and xylem limited pathogens when probed with only the secondary goat anti rabbit Pab conjugated with alkaline phosphatase in the absence of the primary rabbit anti OmpA antibodies (Fig. 8).

## Discussion

The simplicity of tissue printing on nitrocellulose membranes has made it a widely used technique. Others have used immune detection to localize the distribution of rubisco and catalase in tissue sections^27^, of (1–5)-α-L-arabinan, a component of pectin, in leaf sections^28^ and cysteine rich proteins^29^. Enzymatic activities can also be localized to specific locations by tissue printing followed by histochemical detection of the reaction products, for example both pectin methylesterase and acetylesterase in citrus fruits^30^, and peroxidase^31^, and myrosinase activity produced during active defense by plants against pathogens^32^.

Immuno-tissue printing has also been applied to study the distribution of pathogens within plants. Examples include *Apple chlorotic leaf spot virus* and *Apple stem grooving virus* in apple shoots^33^, and *Cucumber vein yellowing virus*^34^. The distribution of RNA associated with pathogens can also be followed by tissue printing using either chemiluminescent or colorigenic assays^35^,^36^. Recently this approach has also been applied to *C*aLas in citrus^37^. Tissue printing has also been applied recently for the detection of *Spiroplasma citri*, another phloem-limited bacterial pathogen of citrus. In this case, rather than detect the pathogen directly, the authors identified a protein secreted by *S. citri* which is transported systemically in the plant. Polyclonal antibodies against this protein provided an effective detection method for the pathogen in infected citrus, in a tissue printing format that is intended to overcome the problem of uneven distribution of the pathogen in the host, a problem that also exists with *Ca*Las^38^.

In the present study we prepared tissue prints from sweet orange and rough lemon tissues that were infected with *C*aLas. The DTBIA was first performed to detect scFv that contained a FLAG epitope at the carboxyl terminus of the scFv. We used scFv that recognized a portion of OmpA, a protein expected to be present in high concentration on the surface of the *C*aLas cells, and FlgI, a component of the flagellar apparatus encoded by *C*aLas^39^. As a control, mock DTBIA was performed with only the secondary anti-FLAG Mab on immuno tissue prints from healthy and diseased petioles. No scFv specific for *C*aLas were used in these DTBIA, yet color development was restricted to the phloem ring as observed previously with the complete assay. Reactions tended to be stronger with diseased samples. Similar detection of the partial FLAG epitope in diseased citrus was also observed with an anti-FLAG Mab obtained from a different commercial source (not shown). Taken together, this suggests that the anti-FLAG Mabs were recognizing a host epitope in the phloem region of *C*aLas infected plants in addition to the FLAG epitope attached to the carboxyl terminus of the scFv.

We therefore performed DTBIA of citrus petioles from trees infected with other systemic pathogens restricted to the vascular system, including mild and severe strains of CTV *(Closteroviridae),* CCDV *(Geminviridae),* and *Xylella fastidiosa*. The pattern of color development was similar in all cases, suggesting that the anti-FLAG Mab was binding to an epitope produced by the plants in response to vascular infection, perhaps a pathogenesis-related protein (PR). Similar levels of purple color were observed by DTBIA in tissue prints made from sweet orange petioles infected with either CTV-B2 (mild strain) or CTV-B6 (severe strain). Therefore the severity of disease symptoms in the plants tested by DTBIA was not a factor in the detection of a partial FLAG epitope in diseased citrus, but the presence of a pathogen was specifically required. The age of the leaves tested was found to be an important factor related to the presence or absence of the partial FLAG epitope in HLB-diseased and healthy samples. When extracts of *C*aLas-infected mature symptomatic leaves were tested by western blotting, much more partial FLAG epitope was found in diseased leaves than in those from healthy controls consistent with the results of tissue printing.

Thus, we have observed a partial FLAG epitope to be present in the phloem tissue in response to several plant pathogens. The DTBIA with the anti-FLAG secondary antibody alone identified plants infected by *C*aLas as well as other vascular pathogens, and may itself be a useful general assay for the presence of *C*aLas and these pathogens. A p-Blast search of the *C. sinensis* proteome identified a paralogous family of proteins that contained the five amino acid sequence DDDDK, which is the carboxy terminal sequence of the FLAG octapeptide. These proteins were present in an SDS-PAGE gel in a region that reacted with the anti-FLAG mAB and were members of the HSP-90/endoplasmin group. HSP-90s proteins play very important role in plant development and are involved in the resistance to pathogens^40^,^41^. Members of the HSP-90 group are secreted chaperone proteins that bind various proteins in response to different developmental programs as well as stress and disease^42^,^43^,^44^. Thus, this group of paralogous proteins has the expected size, biological properties and cellular location as well as the amino acid motif needed to bind the FLAG-antibody in our experiments. No longer sequence identical to the FLAG epitope was observed. The DDDDK sequence was also observed in a single protein of 49.23 kD. This protein is much too small to be responsible for the FLAG epitope that we have observed.

When polyclonal rabbit antibodies against OmpA of *Ca*Las were used in the DTBIA, the assay produced a strong reaction in the phloem ring of petioles taken from HLB diseased, but not healthy tissue prints, as expected. The color development was localized in individual phloem cells as expected for the phloem limited pathogen. In contrast with the results of the scFv detected with the FLAG epitope, no color developed in the phloem cells of tissue prints from healthy plants, or when tissue prints from either healthy or HLB diseased trees were tested with the secondary antibody alone. Also in contrast to the detection of the FLAG epitope, when the anti-OmpA Pab with alkaline phosphatase based detection was tested with tissue prints from trees infected with the other citrus pathogens, the color reaction in the phloem ring was specific for the *C*aLas sample. The goat anti-rabbit Pab did not produce any color reaction at all, as expected, when used in the absence of a primary rabbit antibody.

Thus, the combination of a rabbit polyclonal antiserum against the major outer membrane protein of *C*aLas and a secondary goat anti-rabbit Pab provided specific detection of *C*aLas in the DTBIA. We then tried to use the same anti OmpA polyclonal antibodies in a DAS-ELISA assay for the detection of *C*aLas but without success (not shown). We interpret this to mean that the non-uniform distribution of *C*aLas in phloem tissues provides insufficient density of antigen for the ELISA assay, at the concentration of CaLas found in diseased plant samples (10^7^ genomes/g). However, the natural foci of *C*aLas and the OmpA antigen are retained in tissue prints at much higher local concentrations, providing for a successful detection result. We have shown that this technique can be used to provide very sensitive and detailed information about the distribution of CaLas in citrus petioles, stems, roots, seeds, and fruit^45^. The DTBIA is inexpensive and simple to perform and is readily scalable, and will provide a very useful complement to current PCR based diagnostic tests for *C*aLas. The Hsp-90 secreted chaperone protein family we identified may serve as a useful molecular marker for the diagnosis of cellular pathogens infecting citrus with the convenience of using a commercial anti-FLAG secondary antibody. The evaluation of other antigens for use in similar tissue print applications and the further validation of Hsp-90 proteins as earlier diagnostic molecular marker for high-flux accurate detection of *Ca*Las is currently under investigation in our laboratory.

## Methods

### Plant materials and pathogens

Isolates of *Ca*Las were propagated by bud inoculation onto either rough lemon (*Citrus jambhiri* Lush.) or sweet orange (*Citrus sinensis* L.) seedlings propagated on rough lemon rootstocks. Two isolates of CTV were used: CTV-B2 (very mild symptoms, T30 genotype, Florida) and CTV-B6 (very severe symptoms, VT genotype, California). Isolates of CTV and *Xylella fastidiosa* were maintained by bud inoculation onto ‘Madame Vinous’ sweet orange. *Citrus chlorotic dwarf virus* (CCDV)^46^ was maintained by bud inoculation of alemow (*Citrus x macrophylla* Wester). Trees were grown in Metro Mix 510 potting mix. The greenhouse was maintained at 65–80 °F (18–27 °C) and ambient light was supplemented with high pressure sodium vapor lighting on cloudy days and throughout the winter season to extend the photoperiod. Trees were watered as needed with water containing nitrogen/phosphorus/potassium (100/25/100 ppm), copper (2 ppm) and iron (6 ppm).

### Production and purification of scFv

A library of single chain antibodies (scFv) with 2 × 10^7^ unique members was created using the phagemid vector pKM19 and *Escherichia coli* DH5αF’ for screening and selection, and plasmid pKM16 for propagation of the scFv^21^,^25^. This scFv library was screened to select scFv that bound to segments of proteins expressed on the surface of *C*aLas, including the major outer membrane protein, OmpA (scFv748; YP_003065185) and a membrane component of the flagellar apparatus, FlgI (scFv932; YP_003064789). The sequences that encode these scFv were cloned into the vector pKM16, which was designed to add both a His Tag and a FLAG epitope^47^ to the carboxyl end of the scFv to facilitate purification and screening of the scFv. Single chain antibodies were produced and purified as described^48^ by chromatography with the Ni-NTA purification system (Invitrogen, Frederick, MD). Clones of *E. coli* DH5α (pKM16) with inserts that encoded scFv were inoculated into of LB medium with ampicillin (100 mg/l) and glucose (2%) and grown at 37 °C overnight. Bacteria were diluted 1:10 into fresh media and grown with vigorous shaking until the culture density reached OD_600_ 0.8. The cells were collected by centrifugation at 8000 RPM for 15 min and suspended in 100 ml LB with ampicillin (100 μg/ml), MgCl_2_ (20 mM), and IPTG (1 mM). The bacteria were incubated at 32 °C overnight with shaking and then collected by centrifugation at 8000 RPM for 15 min. The bacterial pellet was suspended in 8 ml of native binding buffer and lysozyme (12 mg) was added followed by incubation on ice for 30 min. The pH of the native binding buffer was adjusted to 8.0 immediately before the purification was started. The cells were broken by six cycles of sonication at high power on ice (Virsonic 300, Virtis Scientific). Each cycle of sonication was for 10 s followed by 30 s of rest. Cellular debris was removed by centrifugation at 8000 RPM and the supernatant was transferred to a clean tube and the manufacturer’s protocol was followed to purify the scFv. Aliquots of the purified scFv were removed for SDS-PAGE and quantification with the Bradford assay (Dojindo Molecular Technologies, Rockville, MD). A proteinase inhibitor cocktail (Sigma P9599) was added and the purified scFv were stored at 4 °C.

### Preparation of polyclonal antiserum

OmpA is a protein found in considerable concentration on the outer membrane of gram negative bacteria. A portion of the gene encoding the major outer membrane protein OmpA of *C*aLas, designated Omp3f, (Supplemental Fig. 1) was cloned into the pET102/D-TOPO vector system for expression in *E. coli* BL21. The expressed protein was purified under denaturing conditions using the 3′ 6X His tag encoded by the vector. SDS-PAGE analysis was used to confirm purity. The purified protein was adjusted to a concentration of 2 mg/ml and used to immunize a New Zealand white rabbit with four injections over a period of 56 days, 0.3 mg protein/injection (Cocalico Biologicals, Reamstown, PA) using TiterMax adjuvant (Sigma Aldrich, St. Louis, MO).

### Direct tissue blot immuno assay

Petioles of symptomatic or asymptomatic leaves of diseased and corresponding healthy greenhouse trees were used to prepare tissue prints, generally following a published protocol^49^. Sections approximately 2 mm thick were cut from petioles and pressed for 10 seconds onto nitrocellulose membranes with 0.45 μm pore size (Whatman, Thermo Fisher Scientific, Pittsburgh, PA). The printed membranes were transferred to Phosphate Buffered Saline with 0.05% Tween-20 (PBST) and washed two times for five minutes each on a reciprocal shaker. The PBST was removed and replaced with SuperBlock (PBS) Blocking Buffer (Thermo Fisher Scientific) for initial blocking at room temperature for 2 h. The membranes were transferred into a second blocking solution (PBST + 5% fat free skim milk) that contained the scFv diluted 1:100 or 1:1000. The tissue prints were incubated for 90 minutes at 37 °C or overnight at 4 °C and washed three times with PBST for 10 min each. Monoclonal antibodies that bind to the FLAG epitope of the scFv and conjugated with alkaline phosphatase (#A9469, Sigma Aldrich, St. Louis, MO) were diluted 1:10,000 and incubated with the tissue print for 1 h at 37 °C. The tissue prints were washed three times with PBST for 10 min each before substrate was added (33 μl NBT + 16.5 μl BCIP; Sigma) in 5 ml of alkaline phosphate assay buffer. Goat Anti Rabbit Pab (#12-448, EMD-Millipore) conjugated with alkaline phosphatase was used to detect Pab bound to OmpA of *C*aLas in tissue print assays. The tissue prints were incubated until color development could be seen. The tissue prints were photographed with a Zeiss Discovery 20 light microscope equipped with a digital camera. Serial sections of petioles were taken for tissue prints, and alternate sections were either used for DTBIA or set aside for extraction of DNA and qPCR to estimate the amount of *C*aLas present in each petiole^50^.

### Identification of a citrus protein that binds anti-FLAG monoclonal antibodies

An antigen that bound anti-FLAG monoclonal antibodies was present in the phloem vessels of diseased citrus. HLB-diseased leaves were removed from sweet orange trees. Petioles were ground in liquid nitrogen and the powder was taken up in buffer. Samples were loaded on 4–15% SDS-PAGE gels (Mini Protean TGX, BioRad Labs, Hercules, CA) and subjected to electrophoresis for 30 min at 60 V and 1 h at 100 V. The resolved proteins were then electro-transferred to nitrocellulose membranes. The nitrocellulose membranes were blocked and treated with anti-FLAG monoclonal antibody and developed as described for the tissue prints. SDS-PAGE gels were also prepared as described but not subjected to electro-transfer. The region corresponding to the region that bound the partial FLAG epitope was cut out of the gel and sent for sequence determination by GeLC-MS/MS with a Q-Extractive Orbitrap device (Bioproximity, Springfield VA). This returned a list of 3999 short peptides (5–53 amino acids) present in the excised SDS-PAGE band of interest. These peptides were blasted against the *C. sinensis* proteome^51^ and the corresponding full length protein for each hit with a negative e-value was retrieved using Bioperl. Proteins between 90–115 kD were searched using the Grep program in Linux for the FLAG peptide sequence and variants.

## Additional Information

**How to cite this article**: Ding, F. *et al*. Serological detection of ‘*Candidatus* Liberibacter asiaticus’ in citrus, and identification by GeLC-MS/MS of a chaperone protein responding to cellular pathogens. *Sci. Rep.* **6**, 29272; doi: 10.1038/srep29272 (2016).

## Supplementary Material

Supplementary Information

## Figures and Tables

**Table 1:** Representative results of TaqMan qPCR for quantification of ‘*Ca*. Liberibacter asiaticus’ in extracts of petioles used for DTBIA.

Host and isolate designation	Cq value^a^	
	FAM (CaLas 16S RNA)	TET (cytochrome oxidase)
CaLas B430 in rough lemon		
B430-1	23.10	19.22
B430-2	21.4	20.67
B430-3	22.64	20.56
B430-4	21.44	20.26
B430-5	22.20	21.03
B430-6	21.60	20.73
CaLas B232 in sweet orange		
B232-1	21.08	19.38
B232-2	21.39	17.37
B232-3	22.58	18.18
B232-4	20.61	19.17
B232-5	20.41	20.85
B232-6	20.21	19.10
**Mean**	21.56 ± 0.92	19.71 ± 1.16

^a^qPCR performed as described (16).

**Table 2:** Peptides identified by GeLC-MS/MS that are part of a HSP-90 protein which contains a portion of the FLAG epitope and is expressed in citrus trees infected with bacterial and viral pathogens^a^,^b^.

>175
VVKDPEDAGVQQTAQLIYQTALMESGFSLNDPKDFASR
>629
VVKDPEDAGVQQTAQLIYQTALMESGFSLNDPKDFASR
>768
GLVDSDTLPLNVSR
>889
VVKDPEDAGVQQTAQLIYQTALMESGFSLNDPKDFASR
>940
VVKDPEDAGVQQTAQLIYQTALMESGFSLNDPKDFASR
>1120
TALMESGFSLNDPKDFASR

^a^Proteins from sweet orange petioles were resolved by SDS-PAGE and submitted for sequence determination by GeLC-MS/MS.

^b^pBlast best matches were to accessions KDO84885.1 and KDO84884.1 and XP_006473673.1 with E values from 3e-07 to 4e-23.

**Table 3:** HSP-90 proteins encoded by the genomes of *Citrus sinensis* and *Citrus clementina* which contain a partial FLAG domain (DDDDK).

Accession	Definition	Amino Acids	Mass kd
KDO84884	Hypothetical CISIN_1g003458 mg [Citrus sinensis]	818	93.88
KDO84885	Hypothetical CISIN_1g003458 mg [Citrus sinensis]	679	78.3
KDO84886	Hypothetical CISIN_1g003458 mg [Citrus sinensis]	762	87.95
XP_006473673	Predicted endoplasmin homolog [Citrus sinensis]	822	94.31
XP_006435195	Hypothetical CICLE_v10000296 mg [Citrus clementina]	820	94.09
XP_006482710	uncharacterized LOC102621298 isoform X1 [Citrus sinensis]	425	49.23
