# Supplementary material for: Serological detection of ‘Candidatus Liberibacter asiaticus’ in citrus, and identification by GeLC-MS/MS of a chaperone protein responding to cellular pathogens
Source: Sci Rep. 2016 Jul 6;6:29272. doi: 10.1038/srep29272 (PMC4933950; doi:10.1038/srep29272)
Supplement: Supplementary Information [file srep29272-s1.pdf]

Serological detection of '*Candidatus* Liberibacter asiaticus' in citrus, and identification by GeLC-MS/MS of a chaperone protein responding to cellular pathogens

Fang Ding[1,2], Yongping Duan[3], Qing Yuan[4], Jonathan Shao[2] and John S. Hartung[2]*

Supplemental Figure 1. Antigen used to immunize mice for the preparation of scFv and rabbit for the preparation of polyclonal sera.

Omp3f (ACT 57245) 751bp

DNA fragment:

ATAGTTCCTATTACTGAAAGCATATCGACAAGTTTTAAGTATGATCTTAGGTTTTTACAATATGGTGCTATATCAGA
AAAAGAAAAGATCCCTTCGATATATACAACGTTAATAGAACATGGAAAATTCAGCAGCCATTCTATTTCCCAAAGTA
TCATCTATAATACACTAGATAACCCAATTGTGCCACGTAAAGGCATGTTGATATCATCTTCTTATGATTATGCAGGT
TTTGGAGGAGATTCTCAATATCATCGGATTGGATCTCGAGCATCGTATTTTTATCTTCTATCAGATGATTCTGATAT
TGTCGGTTCTTTACGATTTGGATATGGATGTGTCATTCCTAGCAATAAAAATTTGCAATTGTTTGATCAGTTCTCAG
TGAGTTCGAATTATTATCTGAGGGGATTTGCATATAAGGGTATAGGTCCGCGTGTGGATAAGAAATATGCGATTGGA
GGTAAGATTTATTCGTCTGCAAGTGCAGCAGTGAGTTTTCCCATGCCTCTTGTTCCTGAAAGGGCTGGTTTGCGTGG
TGCTTTTTTTGTTGATTCTGCGACTCTTTATGCAAATCATGTTGCTCTCGGTGCCGATAAGCTGGAAGGGAATGATT
CTTTCTGGCGTGTTTCTACTGGAGTAGAAATAATGTGGAATTCTCCACTCGGGATGATGGGTGTCTATTATGGTATA
CCATTGCGTCACCGAGAGGGTGATAAAATTCAGCAGTTTGGTTTTCGTATAGGTAAT

Protein:

IVPITESISTSFKYDLRFLQYGAISEKEKIPSIYTTLIEHGKFSSHSISQSIIYNTLDNPIVPRKGMLISSSYDYAG
FGGDSQYHRIGSRASYFYLLSDDSDIVGSLRFGYGCVIPSNKNLQLFDQFSVSSNYYLRGFAYKGIGPRVDKKYAIG
GKIYSSASAAVSFPMPLVPERAGLRGAFFVDSATLYANHVALGADKLEGNDSFWRVSTGVEIMWNSPLGMMGVYYGI
PLRHREGDKIQQFGFRIGN

Expressed fusion protein: 27kDa(OmpA protein)+ 13kDa(His-Patch thioredoxin)+3kDa(V5, 6xHis)=43kDa

This protein was expressed in and purified from *E. coli* BL21 (pET102/DTOPO). The expressed protein was purified using the 3' 6X His tag encoded by the vector (Ni-NTA Purification System, Invitrogen) under denaturing conditions. Mice were immunized with this protein fragment with 100ug Omp3f/injection and a concentration was 500 ug/ml (Dojindo technologies).

Rabbits were injected with same protein fragment Omp3f at concentration of 2 mg/ml, 0.3 mg/injection, four times in 56 days using Titer Max adjuvant (Sigma Aldrich, St. Louis, MO).

Supplemental Figure 2 Amino acid sequences of five citrus proteins that contain a five amino acid sequence similar to the FLAG antigen.

>gi|641866199|gb|KDO84884.1| hypothetical protein CISIN_1g003458mg [Citrus sinensis]

MRKWTIPSILLLLFLVALIPDQGRNIQAKAEDESDKLVDPPKVEEKLGAVPNGLSTDSDVAKREAESISK
RSLRNNAEKFEFQAEVSRLMDIIINSLYSNKDIFLRELISNASDALDKIRFLSLTDKEVLGEGDNTKLEI
QIKLDKEKKILSIRDRGIGMTKEDLIKNLGTIAKSGTSAFVEKMQTSGDLNLIGQFGVGFYSVYLVADYV
EVISKHNDDKQYVWESKADGAFAISEDTWNEPLGRGTEIRLHLRDEAGEYLEESKLKELVKKYSEFINFP
IYIWASKEVDVDVPTDEDDSSDEEEKAEKEEETEKSESESEDEDEDSEKKPKTKTVKETTFEWELLNDVK
AIWLRNPKEVTEEEYAKFYHSLVKDFSDEKPLAWSHFNAEGDVEFKAVLFVPPKAPHDLYESYYNTNKAN
LKLYVRRVFISDEFDELLPKYLNFLKGLVDSDTLPLNVSREMLQQHSSLKTIKKKLIRKALDMIRKIAEE
DPDESTGKDKKDVEKFSDDDDKKGQYTKFWNEFGKSIKLGIIEDAANRNRLAKLLRFESTKSDGKLTSLD
QYISRMKAGQKDIFYITGANKEQLEKSPFLERLKKKNYEVIFFTDPVDEYLMQYLMDYEDKKFQNVSKEG
LKLGKDTKDKELKESFKELTKWWKGALASENVDDVKVSNRLDNTPCVVVTSKYGWSANMERIMQSQTLSD
ASKQAYMRGKRVLEINPRHPIIKELRERVVKDPEDAGVQQTAQLIYQTALMESGFSLNDPKDFASRIYST
VKSSLNISPDAAVEEEDDVEETDADTEMKESSAAKEDVDTEYSGKDEL

>gi|641866200|gb|KDO84885.1| hypothetical protein CISIN_1g003458mg [Citrus sinensis]

MQIKLDKEKKILSIRDRGIGMTKEDLIKNLGTIAKSGTSAFVEKMQTSGDLNLIGQFGVGFYSVYLVADY
VEVISKHNDDKQYVWESKADGAFAISEDTWNEPLGRGTEIRLHLRDEAGEYLEESKLKELVKKYSEFINF
PIYIWASKEVDVDVPTDEDDSSDEEEKAEKEEETEKSESESEDEDEDSEKKPKTKTVKETTFEWELLNDV
KAIWLRNPKEVTEEEYAKFYHSLVKDFSDEKPLAWSHFNAEGDVEFKAVLFVPPKAPHDLYESYYNTNKA
NLKLYVRRVFISDEFDELLPKYLNFLKGLVDSDTLPLNVSREMLQQHSSLKTIKKKLIRKALDMIRKIAE
EDPDESTGKDKKDVEKFSDDDDKKGQYTKFWNEFGKSIKLGIIEDAANRNRLAKLLRFESTKSDGKLTSL
DQYISRMKAGQKDIFYITGANKEQLEKSPFLERLKKKNYEVIFFTDPVDEYLMQYLMDYEDKKFQNVSKE
GLKLGKDTKDKELKESFKELTKWWKGALASENVDDVKVSNRLDNTPCVVVTSKYGWSANMERIMQSQTLS
DASKQAYMRGKRVLEINPRHPIIKELRERVVKDPEDAGVQQTAQLIYQTALMESGFSLNDPKDFASRIYS
TVKSSLNISPDAAVEEEDDVEETDADTEMKESSAAKEDVDTEYSGKDEL

>gi|641866201|gb|KDO84886.1| hypothetical protein CISIN_1g003458mg [Citrus sinensis]

MRKWTIPSILLLLFLVALIPDQGRNIQAKAEDESDKLVDPPKVEEKLGAVPNGLSTDSDVAKREAESISK
RSLRNNAEKFEFQAEVSRLMDIIINSLYSNKDIFLRELISNASDALDKIRFLSLTDKEVLGEGDNTKLEI
QIKLDKEKKILSIRDRGIGMTKEDLIKNLGTIAKSGTSAFVEKMQTSGDLNLIGQFGVGFYSVYLVADYV
EVISKHNDDKQYVWESKADGAFAISEDTWNEPLGRGTEIRLHLRDEAGEYLEESKLKELVKKYSEFINFP
IYIWASKEVDVDVPTDEDDSSDEEEKAEKEEETEKSESESEDEDEDSEKKPKTKTVKETTFEWELLNDVK
AIWLRNPKEVTEEEYAKFYHSLVKDFSDEKPLAWSHFNAEGDVEFKAVLFVPPKAPHDLYESYYNTNKAN
LKLYVRRVFISDEFDELLPKYLNFLKGLVDSDTLPLNVSREMLQQHSSLKTIKKKLIRKALDMIRKIAEE

DPDESTGKDKKDVEKFSDDDDKKGQYTKFWNEFGKSIKLGIIEDAANRNRLAKLLRFESTKSDGKLTSLD
QYISRMKAGQKDIFYITGANKEQLEKSPFLERLKKKNYEVIFFTDPVDEYLMQYLMDYEDKKFQNVSKEG
LKLGKDTKDKELKESFKELTKWWKGALASENVDDVKVSNRLDNTPCVVVTSKYGWSANMERIMQSQTLSD
ASKQAYMRGKRVLEINPRHPIIKELRERVVKDPEVEFFSHFLLTGKDDFSNPNSMYTSLKIC

>gi|568839402|ref|XP_006473673.1| PREDICTED: endoplasmin homolog [Citrus sinensis]
MRKWTIPSILLLLFLLALIPDQGRNIQAKAEDESDKLVDPPKVEEKLGAVPNGLSTDSDVAKREAESISK
RSLRNNAEKFEFQAEVSRLMDIIINSLYSNKDIFLRELISNASDALDKIRFLSLTDKEVLGEGDNTKLEI
QIKLDKEKKILSIRDRGIGMTKEDLIKNLGTIAKSGTSAFVEKMQTSGDLNLIGQFGVGFYSVYLVADYV
EVISKHNDDKQYVWESKADGAFAISEDTWNEPLGRGTEIKLHLRDEAGEYLEESKLKELVKKYSEFINFP
IYIWASKEVDVDVPADEDDSSDEEEKAEKEEETEKSESESESEDEDEDEDSEKKPKTKTVKETTYEWELL
NDVKAIWLRNPKEVTEEEYAKFYHSLVKDFSDEKPLAWSHFNAEGDVEFKAVLFVPPKAPHDLYESYYNT
NKANLKLYVRRVFISDEFDELLPKYLNFLKGLVDSDTLPLNVSREMLQQHSSLKTIKKKLIRKALDMIRK
IAEEDPDESTGKDKKDVEKFSDDDDKKGQYTKFWNEFGKSIKLGIIEDAANRNRLAKLLRFESTKSDGKL
TSLDQYISRMKAGQKDIFYITGANKEQLEKSPFLERLKKKNYEVIFFTDPVDEYLMQYLMDYEDKKFQNV
SKEGLKLGKDTKDKELKESFKELTKWWKGALASENVDDVKVSNRLDNTPCVVVTSKYGWSANMERIMQSQ
TLSDASKQAYMRGKRVLEINPRHPIIKELRERVVKDPEDAGVQQTAQLIYQTALMESGFSLNDPKDFASR
IYSTVKSSLNISPDAAVEEEDDVEETDADTEMKESSAAKEDVDTEYSGKDEL

>gi|567885273|ref|XP_006435195.1| hypothetical protein CICLE_v10000296mg [Citrus clementina]
MRKWTIPSILLLLFLVALIPDQGRNIQAKAEDESDKLVDPPKVEEKLGAVPNGLSTDSDVAKREAESISK
RSLRNNAEKFEFQAEVSRLMDIIINSLYSNKDIFLRELISNASDALDKIRFLSLTDKEVLGEGDNTKLEI
QIKLDKEKKILSIRDRGIGMTKEDLIKNLGTIAKSGTSAFVEKMQTSGDLNLIGQFGVGFYSVYLVADYV
EVISKHNDDKQYVWESKADGAFAISEDTWNEPLGRGTKIRLHLRDEAGEYLEESKLKELVKKYSEFINFP
IYIWASKEVDVDVPTDEDDSSDEEEKAEKEEETEKSESESESEDEDEDSEKKPKTKTVKETTFEWELLND
VKAIWLRNPKEVTEEEYAKFYHSLVKDFSDEKPLAWSHFNAEGDVEFKAVLFVPPKAPHDLYESYYNTNK
ANLKLYVRRVFISDEFDELLPKYLNFLKGLVDSDTLPLNVSREMLQQHSSLKTIKKKLIRKALDMIRKIA
EEDPDESTGKDKKDVEKFSDDDDKKGQYTKFWNEFGKSIKLGIIEDAANRNRLAKLLRFESTKSDGKLTS
LDQYISRMKAGQKDIFYITGANKEQLEKSPFLERLKKKNYEVIFFTDPVDEYLMQYLMDYEDKKFQNVSK
EGLKLGKDTKDKELKESFKELTKWWKGALASENVDDVKVSNRLDNTPCVVVTSKYGWSANMERIMQSQTL
SDASKQAYMRGKRVLEINPRHPIIKELRERVVKDPEDAGVQQTAQLIYQTALMESGFSLNDPKDFASRIY
STVKSSLNISPDAAVEEEDDVEETDADTEMKESSAAKEDVDTEYSGKDEL

>gi|568858337|ref|XP_006482710.1| PREDICTED: uncharacterized protein LOC102621298 isoform X1 [Citrus sinensis]
MGGKSSKHEIAKTKRSSSFHDLHFEVINILTKSFYIRNLVSKKRRRMLVEGYDLDMSYITDRLLAMSFPA
EHMRAVYRNPLWQVKAVLDMRHQEHYKVYNLCIEESYDPEHFYGRVERYPFDDNHVPPLEMIKLLCESVH

SWLSSDPKNIAVIHCMAGKGRTGLMVCSYLVYTGMSAEEALQLYAHKRTTNNEGVSIPSQRRYVGYWNNI
ISFPRGVHSGTPDVNLPKRCSRELLRVRLYDTINTDAVFFVVSELQEVTGQLHRPAMELSRSSCRPVKKG
NQRNTNHKYYVSCIEDEEEGSKLESEEPRVVVQMDTENSIIYQKTCLDHYFDKPLQVSGDVRVIFYQKMI
GSRFFYACFNAAFIRNSMLQFSIRDLDKVGSRGRSICGPSFCLELVFGPANPKCSLVHPHDDDDDDDDDK
HFSHE
